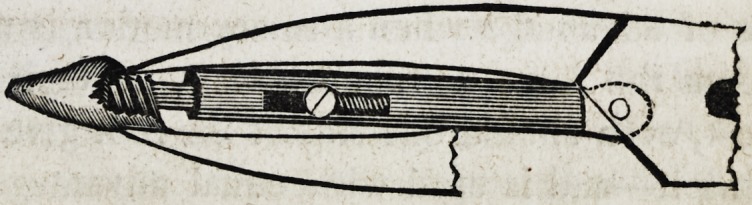# Dental Ethics, and the Compound Screw Forceps

**Published:** 1850-01

**Authors:** C. O. Cone

**Affiliations:** Baltimore, Md.


### ARTICLE IV.

*Dental Ethics, and the Compound Screw Forceps.* By C. O. CONE, M. D., Baltimore, Md.

IN glancing at the police items of our daily papers, one is struck with the frequent occurrence of this notice: "Goods obtained under false pretences." It shows how keenly alive the present generations are to the interests of the purse; how carefully they guard against encroachments on their rights! It is a common fact, however, that almost every other species of imposition is practiced with impunity; "false pretences" of every description are suffered to drive their practical trade undetected and unpunished.

To say nothing of medical quackery, mesmerism, quack literature, and a thousand others of this fellowship—the false pretences of invention and authorship, or quack dentistry, is truly a practical comment on public discernment, not to say "gullibility." Many of these pretences have their origin in mere fortuitous circumstances; but professional duplicities may generally be traced to one of two things, (perhaps both combined:) either a false estimate of our own abilities, or a morbid thirst for *notoriety*—to be applied to means for obtaining gain. This mental disease is becoming quite an epidemic, and the honest few that have not as yet been infected, should be admonished of its incipient approach. To such we would say,—if you possess genius and talents for your profession, and its adornment by invention or improvement—beware of crowding yourself into notice by proclaiming the exclusive invention or practice which is another's, or generally known to the profession, or attempt to palm upon the world the cravings of vanity, in the form of an improvement, which is no improvement at all. Leave to the *honest* and wise among your friends the discovery of your abilities, and the particular sphere of your usefulness, instead of prejudging your own cause; and suffer not self-conceit or injudicious flattery to tempt you to an exposure of weakness which will only render you ridiculous.

Nature's gifts usually develop themselves spontaneously; and the very fact that those gifts are *natural*, is why their possessor is the last to discover anything special in them; they are his unconscious breathings—his involuntary movements—and therefore he needs to learn the fact that the attention of others has been attracted to them. Not so the pretender. He beholds in others a skill and tact for which they are applauded and admired, and he thirsts for the same applause; he attempts to imitate what he admires, and claims for his miserable pretences the credit due real merit. It is the entire absence of a quick sensitiveness and just appreciation of the true and beautiful in talent, which leads to this self-deception, and would strive to reduce a liberal profession to the mean and selfish level of a three dollar patent wooden clock, or other petty articles of traffic.

While placing the above reflections on the subject of dental ethics in a connected form, my attention was directed to two articles that appeared in southern papers, endeavoring to establish certain claims of one Mr. C. H. Dubs to the invention of the "Compound Screw Forceps," and we changed our original design, leaving our manuscript unaltered to head an article in examination of Mr. Dubs' pretensions, leaving readers to apply such of the above truths as may be applicable to the subject now under discussion.

The claims of Mr. Dubs can best be understood by an examination of the publications above referred to, as they originally appeared, as follows:

**[From the Concordia Intelligencer, published at Vidalla, La., for Sept. 29th, 1849.]**

*Dentistry—the North and the South.*

The invention of the dental instrument styled the *Compound Union Screw Forceps*, by Dr. C. H. Dubs, of Natchez, has become the basis of a discussion in the dental journals, which must result beneficially to southern dentists. The great gun of Dentistry—the Baltimore *Journal of Dental Science*, has another communication, (one vindicating the claims of Dr. Dubs,) in its September number; while the New York Dental Recorder and N. Y. Dental News Letter, each have their views on this important dental invention.

These three periodicals are the leading exponents of the dental *"profession"(?)* in the United States. They are singularly unanimous on one important branch of this discussion, viz. that a dentist named Hullihen, two or three years ago, suggested the union of the screw with the forceps in extracting shells of teeth, and gave the benefit of his invention to the Baltimore *Journal of Dental Science;* and that, as now Dr. Dubs has so far improved Hullihen's raw and primitive invention as to make the *Compound Union Screw Forceps* an entirely new instrument, and has preferred to take out a patent therefor, rather than to give it to the "profession," he cannot be a dentist *of the right stripe.*

This "profession" must be the most selfish and anti-progressive concern now existing. It has not yet learned, it would seem, that the only way to advance the cause of science or the practice of dental *physique* is to reward those who show themselves able to advance them. The attempt is idle to unite patriotism and eminence in dentistry in the same aspiration, although, indeed, there can be no patriotism in a dentist inventing an instrument and giving it to other dentists to make money out of it. Ah! but, argue these "Journals of Dental Science" and "Dental Recorders," this instrument of yours, Dr. Dubs, may alleviate much pain, and it is an act of humanity to those suffering to give it to the profession. If they confess this as their position, (and it is hard to say *what* their position is,) just let them reflect what a compliment they thus pay to the members of their profession, in insinuating that they will not *buy* an instrument that alleviates pain; and if this be not their position, by what kind of right does the profession claim the fruits of the genius and science of any one of its members? That *it*, the profession, may decide upon the improvement? The world can judge of the difference between two instruments as well as it can. That it may decide upon the respective dates of inventions competing for priority? The world can do this better, because more impartially, than itself.

We ask these questions after reading the following in the *N. Y. Dental Recorder* for September:

"We are not sure that we exactly understand what Dr. Dubs claims as his invention. We had been led to suppose that it was only a modification of Hullihen's instrument; but from the extract which we publish from a Natchez paper, we should suppose that he claimed the invention of the instrument itself. It is a very easy matter to decide who has the priority of invention in this case, if the claimants are so disposed. The June number of the American Journal of Dental Science for 1844, contains Dr. S. P. Hullihen's description of his "Compound Root Forceps," and we have stated in our July number the time when an Eastern dentist, who had never seen Natchez, nor heard of Dr. Dubs, suggested to us what he thought would be an improvement.

"If Dr. Dubs will inform us what he claims as his invention, and the time when it was invented, established by such proof as will satisfy the profession, we shall take pleasure in publishing it, and placing the matter exactly as it should be with our readers."

Remarking that it is laughable to see such an effort to deny justice to a man, in asking what his patent really is, at this late day, and when the principal dental instrument maker in the United States has been infringing his patent by manufacturing his instruments extensively for a long time past, we would suggest to the *Dental Recorder* that dates and "priority" have nothing to do with the inventions of Dr. Hullihen and Dr. Dubs. They are *two* instruments, and a look at Dr. Hullihen's meagre invention, will satisfy any intelligent man that *he* could not have invented Dr. Dubs' instrument, had his life been at stake. Farthermore, an "Eastern dentist who had never seen Natchez" might very easily have caught the idea from some other "Eastern dentist" who *had* seen Natchez; and Dr. Dubs has written evidence in his possession that "Eastern dentists," or Eastern dental instrument makers, had seen and admired his invention long before the attempt to manufacture one of them was heard of. Our particular object in stating this is to show, that as the "profession" of dentistry seems to have a very nice sense of honor and consistency, it is due to the cherished principles of the profession, at once to condemn the Eastern instrument maker, who takes advantage of information thus communicated, to deprive one of the profession of the fruits of his skill.

But these organs of the "profession" do not choose to do this much justice to a "Southern man," who, with a genius greater than their own, has not yet learned the little *advantages* to be gained by studying the *system of patenting quickly*. The deep grudge against Dr. Dubs is that he did not give the benefit of his invention to *them*. Phew! Dubs would look well in doing so truly;—to *them*,—to men who themselves patent every book that they write, and advertise in every number of their journals "Patent Enameled" inventions, the price of which is "within the reach of those disposed to purchase," and the author of which is "prepared to dispose of licenses under his *patent!*" Does this profession or do these organs of it mean to put their *mark* on the individuals who may with propriety take advantage of our admirable patenting system? It would seem thus.

But Dr. Hullihen's instrument, it appears, cannot be laid aside in deciding upon the merits of the invention of Dr. Dubs. Although, as we before remarked, the one of these instruments is only a very small part of the other, we shall now enable the faithful guardians of the integrity of the dental profession, if they will, to give justice to Dr. Dubs without the trouble to decide on the merits of Dr. Hullihen's instrument.

The Baltimore *Journal of Dental Science* says that its number for June, 1844, "contains Dr. S. P. Hullihen's description of his Compound Root Forceps." This is admitted to be the earliest intimation of the inferior instrument. Now we shall give the word and letter of a man who is as good, where he is known, as the Baltimore *Journal of Dental Science* is anywhere, that the efficient and perfect instrument of Dr. Dubs actually preceded the imperfect skeleton of Hullihen.

[Copy Letter.]

From HARRIS HILL, Esq., of Jefferson Co., Miss., to DR. C. H. DUBS, of Natchez:

JEFFERSON CO., MISS., *Aug.* 30, 1849.

*Dr. Charles H. Dubs, Natchez:*

SIR:—In answer to your inquiry as to my recollection of the time of your first conversation with me on the subject of inventing your Screw Forceps, with a catch, so as to combine the action of the screw and forceps—to the best of my recollection, it occurred in the fall of 1842; and sometime in the year 1843, I think in March, you told me you had commenced making them, or had them completed; and in the fall of 1843 or spring of 1844, you were at my house and offered to extract roots of teeth for my wife, and assured her that operating with your screw forceps would give but little pain. Yours, with respect,

HARRIS HILL.

Dr. Dubs has other letters to prove the same truths. And yet a leading instrument maker in New York has been, for some time, infringing his patent, and manufacturing his instrument under the very noses of the profession, and *they take no notice of it.* Dr. Dubs will do no more than justice to himself in prosecuting the infractor of his patent, and allowing the "profession" to stand aside until some other Southern dentist may invent something that will suit the organs of the said "profession," and prove so undignified as not to *give it to them.*

---

[From the Natchez Courier, Oct. 5, 1849.]

*Dr. Dubs' Patent Compound Union Screw Forceps—the Date of its Invention—the Fallacy of the Doctrine "no Patents to Professional Gentlemen"—Professional Courtesy the surest Passport to Success.*

The northern and eastern dental journals, at first looking sour and scowling upon the invaluable invention of Dr. Chas. H. Dubs, for the safe and easy extraction of a class of the human teeth, heretofore the most difficult and precarious, as well as painful to manage—have of late thrown open their columns to something like fair and unprejudiced statements respecting the value and date of the invention. The progress of truth is slow, but is always sure. Passion may hide facts from view, and malice and detraction deny them for a time; but, at length, like the sun bursting through a cloud, all will be clear and manifest to every body around. So it has been with the inventor of the "Compound Union Screw Forceps." Surgeon dentists would either deny that the instrument was of any use, or, if the use was admitted, they would endeavor to deny that the invention belonged to the inventor in Natchez. Like the Pharisees of old, they were always ready to cry out with holy horror—"Can any good thing come out of Nazareth?" (Natchez.) But "Time, the beautifier of the dead," has, in this case, done justice to the living. Dr. Hullihen's instrument, which was never patented, was announced to the world in 1844; and this is the instrument which it has been charged by slanderous and malicious persons, that Dr. Dubs had seen or heard of, and made the copy of his own invention. But "this pole is too short to knock down the persimmons," for Dr. Dubs has drawings at different dates, long before 1844, showing his various improvements in his instrument, as he perfected it from year to year, until it became so complete and the principle so fully developed, that he sent on to the Patent Office a description and filed his caveat for his invention, some sixteen months before he obtained his patent in October, 1848. The first rough drawing of Dubs' instrument in 1842, is the representation of a more perfect instrument than that of Dr. Hullihen's in 1844, when the latter was promulgated to the world.

The date of the invention does not stand alone on the credit and authority of Dr. Dubs, or his book of drawings.

Mr. John Swain, Jr., long a respected resident of the city of Natchez, and now at the head of a well-known and prosperous manufacturing establishment in New Orleans, writes to Dr. Charles H. Dubs, as follows:

[*Extract.*] "NEW ORLEANS, *Nov. 7th*, 1848.

"DEAR SIR:—It is known to me that you were at work on the forceps in question in the fall of 1842 or the spring of 1843. If it will be of any advantage to you to have my affidavit to this effect, please inform me. You can make what use of this letter you please.

"Respectfully, &c.

"JOHN SWAIN, JR."

The following, from a planter of Jefferson County, of the highest character for veracity and respectability, and well known in Natchez, corroborates the statement of Mr. J. Swain, Jr.

"JEFFERSON CO., MISS., *Aug.* 30, 1849.

*Dr. Charles H. Dubs, Natchez:*

SIR:—In answer to your inquiry as to my recollection of the time of your first conversation with me on the subject of inventing your Screw Forceps, with a catch, so as to combine the action of the screw and forceps—to the best of my recollection, it occurred in the fall of 1842; and sometime in the year 184 , I think in March, you told me you had commenced making them, or had them completed; and in the fall of 1843 or spring of 1844, you were at my house, and offered to extract roots of teeth for my wife, and assured her that operating with your Screw Forceps would give but little pain. Yours, with respect,

"HARRIS HILL."

Besides these conclusive evidences, Dr. J. W. Monette, the historian of the Valley of the Mississippi, is prepared to testify to the date on which the instrument of Dr. Dubs was used in his family, which sets the matter at rest, as to the utter impossibility that Dr. Dubs could have copied from Dr. Hullihen. Indeed, Dr. D. was so careful to do the exactly honorable thing, that, having heard of Hullihen's invention after he had filed his caveat, but before he had obtained his letters patent, he purchased Hullihen's forceps, and sent them on to the patent office at Washington, to show the Commissioner of Patents that he did not claim as his invention a single thing or item belonging to Hullihen's instrument. The instrument thus sent on is now deposited in the patent office, along with the model of the instrument patented by Dr. Dubs.

The instrument invented by Dr. Hullihen, but never patented, has proved so worthless and inefficient, that it has been made by one of the most celebrated instrument makers in New York, with an improvement added, and published for sale as follows: "Hullihen's Screw Forceps with Chevalier's improvement." This "improvement" is nothing more nor less than an imperfect imitation of the patent of Dr. Dubs. It is an innovation of his principle, and will be prosecuted as an infringement upon his patent.

To show how unjust and ridiculous the whim is that it is unprofessional for a surgeon dentist to patent any instrument he may invent, let us take a picture which is no fancy sketch:

An editor or author of a work on Dental Surgery sits down to write an article against the practice of securing patents for any invention that originated in the brain of a Dental Surgeon. He spreads his paper before him, as he seats himself in his patent spring bottom chair; he seizes his patent Perryan pen, dips it into a patent inkstand, filled with Maynard and Noyes' patent ink. He finishes his article; it rains; he draws on his patent water-proof boots, opens his patent umbrella, which is armed with the qualities of a walking-stick and a shelter from sun and shower, and he goes to the printing office, and has his article worked off on a patent power press, and bound up in patent spring back binding. Finally, he secures the patent or copyright to his work! Pish, on such pretensions on the part of a man who is *patent himself all over!*

Dental surgeons will learn, some time or other, that the best proof they can give of their own ignorance and defective education, is to introduce themselves to public notice by endeavoring to write down their professional brethren. The business of a surgeon is to alleviate human woes by his cunning instruments, and the scientific and dexterous mode of handling them—by his good work and unimpeachable and skilful operations. It is not his vocation, to stick his malicious and poison-charged pen under his brother's fifth rib, and grin and mock while he sees "the galled jade wince."

PUT THE SCREWS TO THEM.

---

We will not stop here to examine the sophistry and unprofessional character of the above articles, as they are too plainly visible to need comment; but proceed to lay before the reader Dr. Monette's certificate, which is referred to in the above, and which was evidently given by Dr. Monette in answer to a request, as were the other names which are attached, and which was published in one of the Mississippi papers, shortly after its date. The design of its publication cannot be mistaken, and will be appreciated by the profession.

"Dr. Charles H. Dubs, *Surgeon Dentist:*

"*Dear Sir:*—I have carefully examined your Compound Patent Screw Forceps, for extracting the roots of the incisor and canine teeth, without injury to the alveolar processes. Having seen and personally experienced the superior excellence of this valuable instrument, in some of the most difficult cases, and believing it one of the most complete instruments for that purpose which has ever been invented, I should have no hesitation in recommending it to the medical profession generally, and specially to those who are practical operators in dentistry. It is the very instrument which has been so long wanted, and which should certainly be in the hands of every dental operator who aspires to perfection in the practical advantages of his profession. Its great excellence consists in the admirable *union* of the screw and forcep, thereby greatly augmenting the powers of each in the extracting of roots which would yield to *neither separately;* and that without the slightest injury to the alveolar process preparatory to the skilful adaptation of artificial teeth set on plate. Indeed its superior excellence is duly appreciated only after the frequent successful use of it in cases which foil the most skilful operator with any other instrument.

"Very respectfully,

"J. W. MONETTE, M. D.

"Washington, Miss., *March* 20, 1848."

"We, the undersigned, agree with the sentiments of Dr. J. W. Monette's description of the above described patent instrument.

A. G. THORNTON, M. D.
C. H. STONE, M. D.
H. LYLE, M. D."

We hazard little in expressing the opinion, that Dr. Monette had no acquaintance with Dr. Hullihen's forceps, or that such an instrument had been in use by the profession for some years, at the time he gave his approval to the "Patent Screw Forceps;" and which opinion Mr. Dubs *very modestly permits* to be published, with three other endorsements; informing the public, in language not very indirect, that Mr. Dubs is *the* dental surgeon who possesses the instrument for extracting fangs of teeth with little pain, "and that without injury to the alveolar processes preparatory *to the skilful adaptation of artificial teeth set on plate.*" Indeed, all that has appeared from Mr. Dubs, in relation to his instrument, has borne this feature—that he regarded his instrument in the light of a mercantile speculation, and a favorable medium of publicity.

Before we go further, we would desire to prevent any misunderstanding, and disclaim having any sectional prejudice or personal interest in the matter, other than a desire to restore to Dr. Hullihen justice, which, it would appear to us, duplicity had attempted to deprive him of.

There can be no question in relation to the extent or character of Mr. Dubs' pretension, as expressed in the above quotations—which, so far as the screw forceps are concerned—he claims without reserve all and every part of the invention. This presents a singular contrast with language used in other places, and on other occasions.

In a number of the Eureka, a record of mechanism, inventions, patents, science and news, published (I think) in the month of March, 1849, Mr. Dubs expressly denies claiming any other part of the screw forceps as his invention, other than the *ratchet, click and spring.*

An article published in the July number, 1849, of the American Journal and Library of Dental Science, and from the pen of a friend of Mr. Dubs, who no doubt wrote by authority, also says, "Dr. Dubs expressly claims as follows, in his specification in his patent, No. 5865: '*A combination of springs and notches on the shaft of the screw, with the catch of the click, by means of which the screw affords additional power in extracting roots of teeth.*'" Again, the same is repeated in the October number, 1849, of the Dental News Letter, p. 12, over the signature of C. H. Dubs, and in the following language: "The following precise words, taken from my specification, and now in my letters patent, *show all that I claim as my invention:* 'What I claim as my invention, and desire to secure by letters patent, is the combination of the notches of the screw with the catch of the click, by means of which the screw affords additional power in extracting roots of teeth, as above described.'"

By a comparison of the last quotation with Mr. Dubs' pretensions, set forth in the articles published in the newspapers of Sept. 19th and Oct. 5th—the three articles appearing almost simultaneously—presents a strange contrast of language, and a species of professional legerdemain which does not demand acute perceptive faculties to detect.

When Mr. Dubs wrote his articles for his southern and undoctrinated readers, claiming as his invention the whole of the instrument under consideration, he could hardly have forgotten that he had written an article a few days before, for a professional periodical, which was likely to meet the eye of those who were familiar with some of the facts; claiming only a *small part* of the instrument as his invention! But if so, it is fortunate that other evidence exists in the case than that furnished by Mr. Dubs' staggering and accommodating memory.

Perhaps Mr. Dubs may say, that he was not "careful to forget what he remembered" on certain occasions, and that the articles published at the South, in newspapers, were the productions of friends, for which he does not feel himself held responsible. To this we would reply, that Mr. Dubs gave the articles now in question his countenance, if not authority—first, by procuring and furnishing letters designed to sustain the claims set up in the communications; and secondly, in failing to publicly contradict these misstatements when published.

We will now endeavor to give a plain statement of the facts which mark the history and invention of the Compound Screw Forceps. The first instrument that was made, was manufactured by Mr. William Kryter, instrument maker, Wheeling, Va., in the month of October, 1838, as shown by the following letters. During the same year, Mr. Kryter made instruments of the same pattern for a number of dentists who passed through Wheeling, and some of them on their way *South*.

The following is in answer to a letter addressed to

Dr. Hullihen, making inquiry when he first invented the Compound Screw Forceps:

"WHEELING, *Oct.* 1*st*, 1849.

"PROF. CONE:

"*Dear Sir:*—In answer to your inquiries, as to the time I first contrived the Compound Screw Forceps, I must say, that I was under the impression ever since Dubs took out a patent for this instrument, that it was in the year 1842 that I had the first instrument of this kind made, and have so stated to some of my friends; but I find, upon looking over the books of my instrument maker, Mr. Kryter, that I was charged with the first pair "Oct. 10th, 1838." This date will show, as near as possible, the time the instrument was first invented. Very respectfully, yours,

"S. P. HULLIHEN."

The following letter was received in answer to inquiries made of Mr. Kryter, when he manufactured the first pair of Compound Screw Forceps for Dr. Hullihen:

"WHEELING, *Oct.* 27*th*, 1849.

"PROF. C. O. CONE:—

"In answer to your inquiries as to the time Dr. Hullihen's Compound Screw Forceps were first made by me, I find, by reference to my books, that the Dr. is charged with the first pair, Oct. 10th, 1838; shortly after this, I made him a second pair—and then again, in the summer of 1839, I made him two pairs more.

"The forceps to which I refer, are the same as are represented by cuts in the June number of the 'Journal of Dental Science' for the year 1844.

"Most respectfully yours,

"WILLIAM KRYTER

"*Surgical Instrument Maker.*"

During the winter of 1842–3, Dr. Hullihen visited the East, and while there he described the Compound Screw Forceps to several instrument makers, among whom was Mr. F. Arnold, of this city. In June, 1843, as Prof. C. A. Harris was passing through Wheeling, he called at the office of Dr. Hullihen, where he saw the Compound Screw Forceps, and advised Dr. Hullihen to publish a description of the same, which he did in the June number, 1844, of the American Journal and Library of Dental Science. The plates and description of the instrument, then published, we give below, as it will enable us to understand what merit is to be attached to Mr. Dubs' improvement, if any.

"*Compound Root Forceps.* By DR. S. P. HULLIHEN, Wheeling, Va.

"The above named forceps were contrived some time since, for the purpose of extracting hollow roots of teeth, with more expedition, and at the same time with less pain to the patient, than was possible with the instruments in general use; and as the forceps have fully answered the purpose for which they were intended, I have thought them of sufficient importance to lay them before the profession.

"The Compound Root Forceps are about nine inches in length, and like the common straight forceps, with the exception that the beak is much longer, and much narrower and thinner at the point. Lengthwise, within and between the blades of the beak is a steel tube, one end of which is open—the other solid and flat, and jointed in a mortice in the male part of the forcep's joint. When the forceps are opened, this joint permits the tube to fall backwards and forwards from one blade of the beak to the other, without any lateral motion. Within this tube is a spiral spring which forces up a shaft—two-thirds of the length of the shaft is rounded and fitted neatly into the tube; the other part is a well tapered or conical screw. The shaft is retained in the tube by a small screw, that is fixed into the shaft through a notch half an inch long in one side of the tube. The shaft and tube are so fitted together, and to the beak of the forceps, that one-half of the rounded part of the shaft projects beyond the end of the tube; so that the shaft may play up and down upon the spring the length of the notch, and the screw part projecting beyond the point of the forceps, so that the shaft may be embraced between its blades, just behind the base of the screw. A full sided view of the beak of the forceps, with its tube and shaft, is well represented in the cuts.

"The forceps are used by first embracing the shaft between the blades; then screwing it gently, and as deeply into the root as possible, the blades are opened—pushed up upon the root, which is then seized in the manner represented in the annexed cut.

"The screw thus combined with the forceps, prevents the root from being crushed. It acts as a powerful lever when a lateral motion is given; it is likewise of advantage when a rotary motion is made—it prevents the forceps from slipping, or of their action being lost, should even one side of the root give way in the act of extracting it—and is used with equal advantage where one side of the root is entirely gone. In short, this combination of the screw and forceps forms an instrument which fulfils every indication that can be desired in the extraction of hollow roots.

"The shaft of the Compound Root Forceps is easily changed; a number of different sized screws may therefore be used in the same pair of forceps."

The only improvement that Mr. Dubs can claim as having made in the Compound Screw Forceps, as shown above, is an addition, but no improvement at all. He has added a ratchet to the shaft of the screw, and a click with a spring bearing against the same. This addition was made with a mechanical eye, and without a correct surgical knowledge of the method of performing the operation for which the instrument was designed. The instrument as invented by Dr. Hullihen, and shown above, secures to the operator all the power that is attained by Mr. Dubs, for the proper extraction of the superior single fanged teeth. The "screw acts as a powerful lever, when a lateral motion is given; it is likewise of advantage when a rotary motion is made—it prevents the forceps from slipping, or their action being lost, even should one side of the root give way in extracting it—and is used with equal advantage when one side of the root is gone." The force applied for the extraction of the superior anterior teeth, is a lateral and rotary or anterio-posterior motion, and not a pull on a straight line with the long axis of the fang, as Mr. Dubs' addition would seem to require; and if so, the improvement or addition indicates that the inverter was not familiar with the proper application of the force for the correct extraction of the teeth to which the Compound Screw Forceps are designed to be applied.

## Figures and Tables

**Figure f1:**
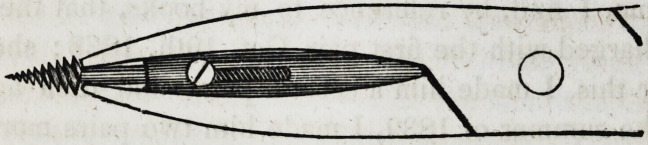


**Figure f2:**